# Supplementary material for: Seminoma and Embryonal Carcinoma Footprints Identified by Analysis of Integrated Genome-Wide Epigenetic and Expression Profiles of Germ Cell Cancer Cell Lines
Source: PLoS One. 2014 Jun 2;9(6):e98330. doi: 10.1371/journal.pone.0098330 (PMC4041891; doi:10.1371/journal.pone.0098330)
Supplement: File S1 — ZIP file containing DMRforPairs output for significant regions. Please start from the html files. (ZIP) [file pone.0098330.s008.zip › figures/39032.pdf]

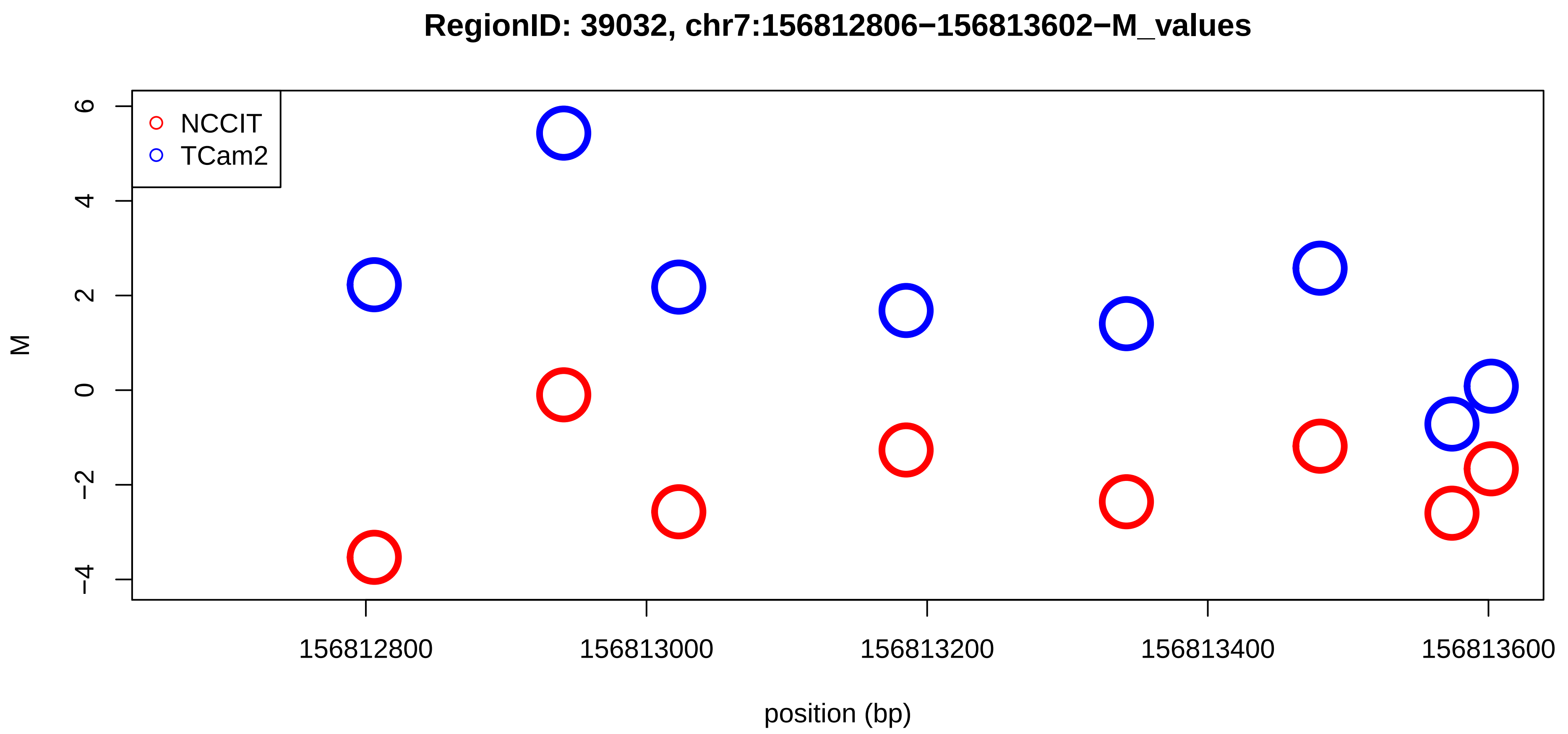

RegionID: 39032, chr7:156812806−156813602−M_values
M
position (bp)
NCCIT
TCam2
6
4
2
0
−2
−4
156812800
156813000
156813200
156813400
156813600

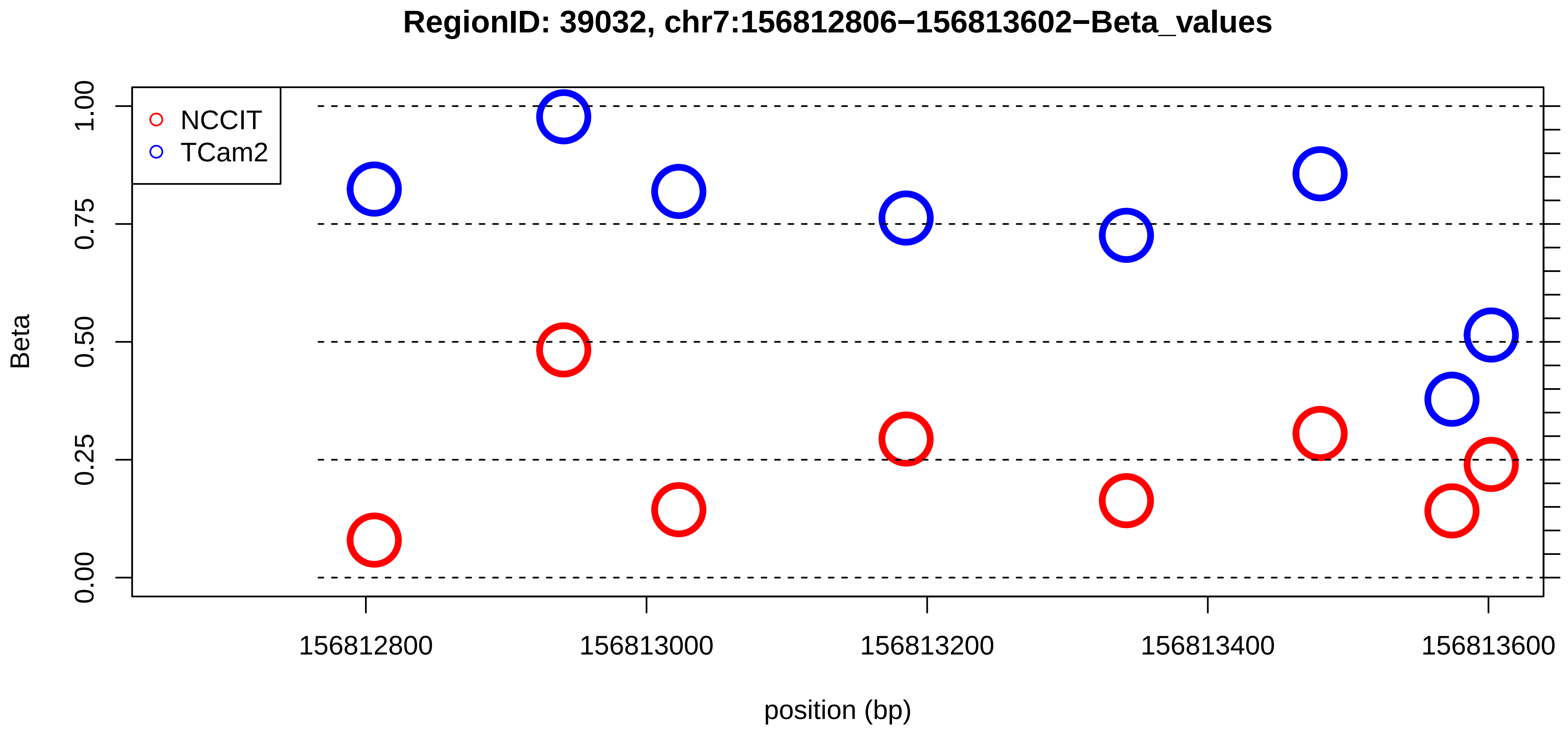

RegionID: 39032, chr7:156812806−156813602−Beta_values
Beta
position (bp)
NCCIT
TCam2
1.00
0.75
0.50
0.25
0.00
156812800
156813000
156813200
156813400
156813600
